# Medical management of post-phacoemulsification uveal effusion in nanophthalmos: A case report^☆^

**DOI:** 10.1016/j.ajoc.2025.102507

**Published:** 2025-12-29

**Authors:** Konstantinos Tirlis, Thomas Chontos, Menelaos Kanakis, Petros Petrou, Ilias Georgalas

**Affiliations:** a 1st University Clinic of Ophthalmology, General Hospital of Athens "G. Gennimatas", National and Kapodistrian University of Athens, Athens, Greece; b Patras University Eye Clinic, Greece

**Keywords:** Nanophthalmos, Uveal effusion syndrome, Exudative retinal detachment, UES, RD, Retinal detachment

## Abstract

**Purpose:**

Nanophthalmos presents a unique surgical challenge due to anatomical constraints and increased risk for postoperative complications. In presenting this case, our aim is to raise awareness of nanophthalmic uveal effusion syndrome (UES) and exudative retinal detachment (RD), emphasize the importance of accurate postoperative diagnosis in such cases, and challenge the prevailing notion that surgical intervention is required in such scenarios.

**Observations:**

We report a case of successful non-surgical management of UES with exudative RD following uncomplicated cataract surgery in a nanophthalmic eye. A 65-year-old monocular female with nanophthalmos (axial length 15.25mm) underwent phacoemulsification with intraocular lens implantation. Postoperatively, the patient developed cystoid macular edema, and subretinal fluid. Prominent choroidal folds and the absence of retinal breaks aided the diagnosis of UES with exudative RD. Given the complexity of this case, and the patient's monocular status, we opted for a conservative approach. A regimen of topical dexamethasone and oral acetazolamide was initiated. Clinical improvement was observed within days, with complete resolution of subretinal fluid and reattachment of the retina over the following month. Six months postoperatively, best-corrected visual acuity improved to 6/12, with maintained anatomical and functional stability at one year.

**Conclusions and Importance:**

This case highlights the importance of accurate postoperative assessment in nanophthalmic patients, particularly distinguishing exudative from rhegmatogenous RD. Furthermore, it underscores the potential for conservative therapy in managing UES, challenging the prevailing reliance on surgical intervention. Further research is needed to delineate criteria for medical management candidacy and optimize treatment regimens.

## 1 Introduction

Nanophthalmos is defined by a reduced axial length (AL) (<21 mm) owing to a shortened anterior and posterior chamber, without additional malformations.^1^ Associated morphological alterations, include a thickened sclera, compressing and impeding vortex veins drainage, resulting in a thickened and congested choroid. Congested choriocapillaris leakage can lead to uveal effusion syndrome (UES) and exudative Retinal Detachment (RD).^2^^,^^3^ The small anterior segment makes cataract surgery challenging, with increased risk of complications and poorer outcomes.^4^, ^5^, ^6^ This case highlights a successful non-surgical approach to post-phacoemulsification nanophthalmic UES with exudative RD.

## 2 Case report

**Fig. 1:**
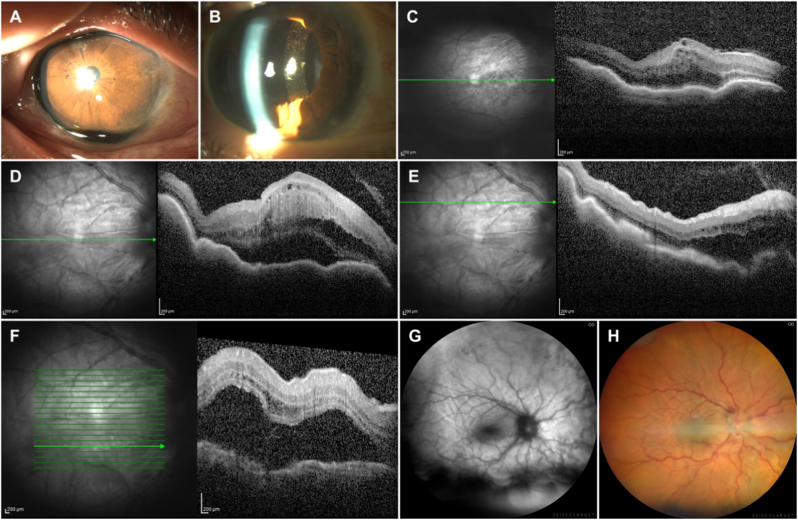
**A:** Preoperative image of the anterior segment of the right eye, with visible iridotomy and shallow anterior chamber. **B:** Postoperative image of the anterior segment of the right eye (4th post-op day), with clear cornea, quiet and deep AC, well-positioned IOL with iris pigment deposition on its surface. **C:** OCT (4th post-op day) revealing cystoid macular edema and subretinal fluid **D, E, F:** OCT (9th post-op day) thickened and detached retina with intraretinal fluid and prominent choroidal folds. **G,H:** Autofluorescence and color fundus images, depicting the shallow retinal detachment and choroidal folds. (For interpretation of the references to color in this figure legend, the reader is referred to the Web version of this article.)

A 65-year-old female presented with progressively worsening vision in her right eye (OD). Slit lamp examination in OD revealed a shallow anterior chamber (AC), a non-dilating pupil with a patent iridotomy, 360° posterior synechiae, and dense nuclear and posterior subcapsular cataract (Fig. 1A). In the left eye (OS), we note an equally shallow AC with prolapsed silicon oil, aphakia, and a total retinal detachment.

Ocular history revealed phacoemulsification without IOL implantation in OS, followed by unsuccessful vitrectomy with silicon oil tamponade for RD. Best Corrected Visual Acuity (BCVA) was 6/120 in OD and no light perception (NLP) in OS. Intraocular pressure (IOP) measured 15 mmHg and 10 mmHg, respectively.

Consequently, the patient was scheduled for cataract surgery in OD. Biometry confirmed nanophthalmos, with an AL of 15.25 mm, a very shallow AC (1.86mm) and R1/R2 values of 6.77/6.70 mm respectively. A +60D IOL was selected for implantation according to the Hoffer Q formula.

The fourth day after an uncomplicated surgery, BCVA was 6/20 (+1.00 D), with a clear cornea, deep and quiet AC, narrow angles (Schaffer Grade 2), well-positioned IOL and attached retina (Fig. 1B), though OCT revealed cystoid macular edema and Subretinal Fluid (SRF) formation (Fig. 1C). The patient returned 5 days later complaining of sudden loss of vision. BCVA was reduced to Hand Motion, AC was deep and quiet and IOP was 20 mmHg. Fundoscopy revealed a shallow, mobile macula-off inferior RD, though no retinal hole or tobacco dust could be discovered. Ultrasonography revealed a shallow serous RD, along with thickened and detached choroid. OCT also depicted a thickened and detached retina with intraretinal fluid and prominent choroidal folds. (Fig. 1D–F). These findings were also evident in both autofluorescence and color fundus images (Fig. 1G–H).

**Fig. 2:**
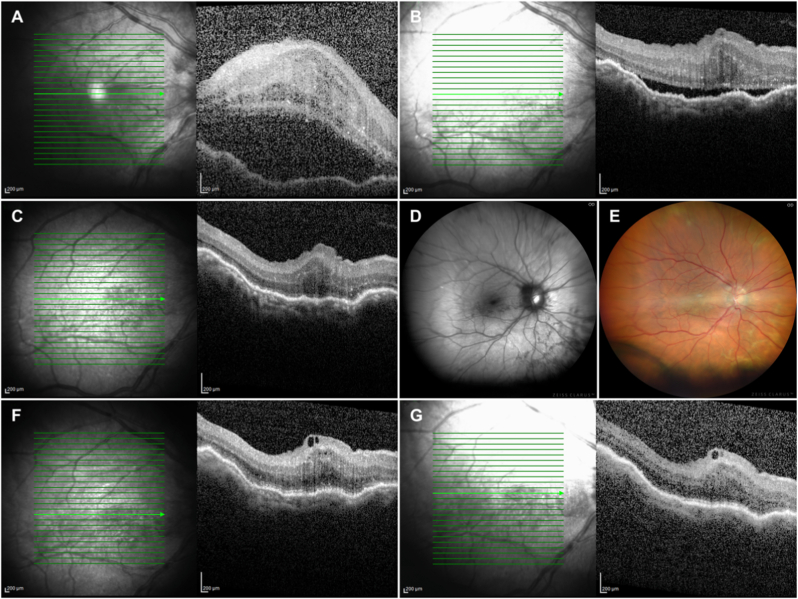
**A:** OCT (24th post-op day/15 days post UES) with persistent subretinal fluid and macular edema. **B:** OCT (31st post-op day/22 days post UES) abrupt reduction of both subretinal fluid and macular edema. **C:** OCT (38th post-op day/29 days post UES) complete resolution of subretinal fluid with residual cystic spaces in the outer retina. **D,E:** Autofluorescence and color fundus image respectively, revealing resolution of the retinal detachment and less prominent choroidal folds. **F,G:** Follow-up (6 and 12 months post-op), with attached macula and dissapearence of the outer retina cystic spaces. (For interpretation of the references to color in this figure legend, the reader is referred to the Web version of this article.)

The diagnosis of UES with serous retinal detachment was established, and treatment with local dexamethasone (q2h) and PO acetazolamide (250mg TID) was initiated. We noted a positive response to treatment, as SRF gradually decreased over a week (Fig. 2A), followed by rapid resolution within the next week (Fig. 2B), at which point local dexamethasone was reduced to q4h for a week followed by QID dosing for another week. Complete SRF resolution and retinal reattachment occurred at one month. Residual cystic spaces still persisted in the outer retinal layers (Fig. 2C). Choroidal folds also significantly subsided as depicted in autofluorescence and color fundus imaging (Fig. 2D–E). We then initiated a careful tapering of local dexamethasone and oral acetazolamide over the following month. At 6 months postoperatively, the patient achieved a BCVA of 6/12, while macula remained attached and outer retinal cystic spaces disappeared (Fig. 2F). At the latest follow-up, one year later, retina was attached and BCVA remained stable at 6/12.

## 3 Discussion

Cataract surgery in nanophthalmic eyes is particularly challenging. These eyes are deeply set, highly hypermetropic, with steeper corneas of reduced diameter (<11 mm).^1^ Altered corneal biomechanics, anterior segment crowding, and thicker lenses contribute to elevated IOP and angle closure glaucoma risk.^1^^,^^7^ The thickened and inelastic sclera predisposes to choroidal congestion, uveal effusion, and serous RD, while malformations of the foveal avascular zone also impact on visual prognosis.^1^^,^^8^^,^^9^Thorough pre-operative evaluation of the posterior segment is highly recommended.^10^

The frequency of post-phacoemulsification UES in nanophthalmic eyes varies across published case series.^5^^,^^11^ Intra and post-operative IOP fluctuations may play a critical role in the pathophysiology of uveal effusion.^12^ IOP elevations compress vortex veins inside their scleral tunnel (elongated in nanophthalmic eyes).^13^ Conversely, hypotony (especially a rapid intraoperative IOP decrease) can hinder pressure-dependent venous outflow precipitating UES,^3^^,^^13^ increasing the risk of RD, vitreous hemorrhage, or malignant glaucoma.^14^^,^^15^ Notably, surgical inflammation is a well-recognized risk factor for UES.^3^

Apart from minimizing intra-operative IOP fluctuations,^15^ no other well documented or unanimously accepted preventive measures exist. Prophylactic steroids or acetazolamide seem to be ineffective.^5^ Although, prophylactic sclerostomy seemingly reduces the risk of UES,^16^ it carries the potential risk of uveal prolapse.^15^

Differentiating exudative from rhegmatogenous RD is critical and surgeons must remain vigilant for the risk of UES and exudative RD in the postoperative period. This can be challenging as mydriasis is usually insufficient, and both scleral indentation and peripheral retinal examination with contact lenses may not be feasible. The presence of choroidal folds and the absence of tobacco dust are useful clinical markers in favor of UES. A critical aspect of this case was the misdiagnosis of uveal effusion in the contralateral eye. Communication with the previous surgeon revealed that vitrectomy with silicone oil tamponade had been erroneously performed under the false assumption of a rhegmatogenous detachment following phacoemulsification. This highlights the potential for devastating consequences when uveal effusion is misdiagnosed.

As UES is extremely rare and standardized treatment algorithms are currently lacking. Most studies advocate surgical approaches, reporting favorable outcomes (primarily with full-thickness or subscleral sclerectomy, but also vortex vein decompression through unroofing, sclerotomy without sclerectomy, and combined approaches).^3^^,^^17^ However, surgery can be technically demanding, and associated with a high risk of complications, including scleral ectasia, traumatic expulsive hemorrhage, infection, and hypotony.^17^^,^^18^

Despite the prevailing notion that UES medical management is usually unsuccessful,^3^ recent literature documented several UES cases that responded to conservative treatment.^17^^,^^19^^,^^20^ Treatment options include local and/or systemic corticosteroids, topical prostaglandin analogues, nonsteroidal anti-inflammatory drugs (NSAIDs), systemic carbonic anhydrase inhibitors, anti-VEGF injections, or a combination thereof. Li et al.^17^ compiled multiple case reports and small case series, demonstrating the efficacy of non-surgical approaches in UES management. However, they identified only two reports of nanophthalmic UES treated successfully with medical therapy alone.

Given the complexity of the case and the patient's monocular status, we opted for a conservative treatment strategy with a potentially safer profile. Oral acetazolamide was initiated with the dual intent of accelerating the resorption of subretinal and cystoid macular fluid by stimulating retinal pigment epithelium (RPE) fluid transport,^21^, ^22^, ^23^, ^24^ and of further reducing intraocular pressure (while avoiding hypotony) to enhance trans-scleral and choroidal venous outflow in the nanophthalmic eye.^1^

Topical dexamethasone was added to the treatment scheme to address the UES surgical inflammation component. Previous reports and reviews have described variable responses to topical, periocular, and systemic corticosteroids across different UES subtypes.^1^ Some evidence supports the efficacy of oral corticosteroids in managing various forms of UES. Nonetheless, successful treatment of non-nanophthalmic cases with topical corticosteroids has also been reported.^25^ We anticipated that a topical regimen might adequately address the surgically induced inflammatory component of UES in this nanophthalmic eye. An early response to treatment (within three days), encouraged the continuation of the regimen, with subsequent complete resolution of subretinal fluid at two months, while retina remained attached 6 months postoperatively.

Finally, it must be mentioned that retinal cystic changes are a recognized feature of nanophthalmos. These changes can involve nearly any retinal layer but most often affect the inner nuclear and ganglion cell layers, with occasional extension into the outer retina.^26^ In our case, cystic spaces were initially concentrated in the inner nuclear layer, later enlarging, and extending into the outer plexiform layer. The progressive nature of the edema enabled us to classify the findings as CME. The near-complete resolution of these changes on long-term follow-up further supports this interpretation. Nevertheless, characteristic nanophthalmic alterations should remain part of the differential diagnosis when evaluating CME.

This case represents an instance of successful non-surgical management of post cataract surgery UES with exudative RD in a nanophthalmic eye, using local dexamethasone and oral acetazolamide. However, larger case series and case-control studies are necessary to further assess the efficacy of medical therapy and the specific regimen across different clinical scenarios.

## 4 Conclusions

This case highlights the importance of postoperative vigilance in nanophthalmic eyes following uneventful cataract surgery and the importance of differentiating between post-operative UES with exudative RD and rhegmatogenous RD. Misdiagnosis can lead to unnecessary surgical intervention with devastating consequences. Moreover, while surgical intervention remains the mainstay UES treatment, emerging evidence suggests that medical therapy may be effective in select cases, avoiding unnecessary surgical risks.

## CRediT authorship contribution statement

**Konstantinos Tirlis:** Writing – review & editing, Writing – original draft, Supervision, Project administration, Conceptualization. **Thomas Chontos:** Writing – review & editing, Writing – original draft, Data curation. **Menelaos Kanakis:** Writing – review & editing, Visualization. **Petros Petrou:** Writing – review & editing, Supervision. **Ilias Georgalas:** Writing – review & editing, Validation, Supervision, Project administration, Methodology, Conceptualization.

## Patient consent

Consent to publish this case report has been obtained from the patient in writing. This report does not contain any personal identifying information.

## Authorship

All authors attest that they meet the current ICMJE criteria for Authorship.

## Funding

The authors received no funding in relation to this work.

## Declaration of competing interest

The authors declare that they have no known competing financial interests or personal relationships that could have appeared to influence the work reported in this paper.
